# The emerging role of neutrophilic extracellular traps in intestinal disease

**DOI:** 10.1186/s13099-022-00497-x

**Published:** 2022-06-22

**Authors:** Feng Chen, Yongqiang Liu, Yajing Shi, Jianmin Zhang, Xin Liu, Zhenzhen Liu, Jipeng Lv, Yufang Leng

**Affiliations:** 1 The First Clinical Medical College of Lanzhou University, Lanzhou, 730000 Gansu People’s Republic of China; 2 Department of Anesthesiology, First Hospital of Lanzhou University, Lanzhou, 730000 Gansu People’s Republic of China

**Keywords:** Intestinal diseases, Neutrophils, Neutrophil extracellular traps, Infection, Inflammation, Colitis, Therapeutics, Colorectal neoplasms

## Abstract

Neutrophil extracellular traps (NETs) are extracellular reticular fibrillar structures composed of DNA, histones, granulins and cytoplasmic proteins that are delivered externally by neutrophils in response to stimulation with various types of microorganisms, cytokines and host molecules, etc. NET formation has been extensively demonstrated to trap, immobilize, inactivate and kill invading microorganisms and acts as a form of innate response against pathogenic invasion. However, NETs are a double-edged sword. In the event of imbalance between NET formation and clearance, excessive NETs not only directly inflict tissue lesions, but also recruit pro-inflammatory cells or proteins that promote the release of inflammatory factors and magnify the inflammatory response further, driving the progression of many human diseases. The deleterious effects of excessive release of NETs on gut diseases are particularly crucial as NETs are more likely to be disrupted by neutrophils infiltrating the intestinal epithelium during intestinal disorders, leading to intestinal injury, and in addition, NETs and their relevant molecules are capable of directly triggering the death of intestinal epithelial cells. Within this context, a large number of NETs have been reported in several intestinal diseases, including intestinal infections, inflammatory bowel disease, intestinal ischemia–reperfusion injury, sepsis, necrotizing enterocolitis, and colorectal cancer. Therefore, the formation of NET would have to be strictly monitored to prevent their mediated tissue damage. In this review, we summarize the latest knowledge on the formation mechanisms of NETs and their pathophysiological roles in a variety of intestinal diseases, with the aim of providing an essential directional guidance and theoretical basis for clinical interventions in the exploration of mechanisms underlying NETs and targeted therapies.

## Background

Neutrophils are the most abundant immune cells and the fastest recruitment cells in infected or inflammatory sites, so they constitute the first line of immune defense of the human body. They have a variety of immune functions, including phagocytosis, production of reactive oxygen species (ROS), degranulation and formation of NETs [1]. NETs have been originally described as the host defense mechanism that used to capture or kill pathogenic microorganisms for neutrophils. It is a kind of reticular structure where neurophils are released to extracellular after stimulation and activation. With DNA as its skeleton, it is embedded with proteins such as histone (histone, H), myeloperoxidase (myeloperoxidase, MPO), neutrophil elastase (NE), cathepsin G (CG) and protease 3 (PR3) and so on, which have bactericidal and permeability-increasing effects and some of them were modified after transformation in the process of forming NETs [2, 3]. In fact, inhibition of NETs formation increases the sensitivity of mice and humans to bacterial infections [4, 5]. However, increasing evidence shows that NETs also contribute to the aggravation of inflammation [6], the occurrence of autoimmune diseases [7], the metastasis and development of cancer [8] and so on. Due to new detection and imaging methods, the exploration of NETs have expanded from in vitro observation to in vivo organ level, and engaged in many specific disease areas, such as systemic lupus erythematosus, vasculitis, diabetes, thrombosis and lung injury [9–12]. Recent studies have found that NETs were related to intestinal diseases. On the one hand, it can prevent bacterial translocation and promote the repair of intestinal mucosal injury and plays an important role in maintaining the stability of intestinal epithelium [13]. On the other hand, excessive NET formation can also destroy intestinal mucosal barrier function, damage intestinal epithelium, and play a key role in the pathological process of a variety of intestinal diseases [6, 14]. Therefore, in this review, we describe the latest findings regarding NETs related to intestinal infection, intestinal inflammation, inflammatory bowel disease (IBD) and cancer and we hope to clarify the disease mechanism of drug treatment and develop new diagnosis and treatment strategies.

## Neutrophilic Extracellular Traps (NETs)

In 1996, Takei H first described NETs as a new type of programmed cell death, which was different from apoptosis with nuclear pyknosis and cytoplasmic vacuolization, and different from cell necrosis that maintains the integrity of nuclear membrane. It’s a special mechanism of cell lysis and death, in which neutrophils show morphological changes, nuclear membrane rupture, nuclear components released into the cytoplasm, and finally the plasma membrane broken, resulting in the formation of NETs outside the cell [15]. In 2004, Brinkmann V redefined NETs as a microbial mechanism involved in cell death, namely activated neutrophils amplify the effectiveness of their antibacterial particles by producing a large network of DNA fibers wrapped in protein particles in a concentrated area, which contributes to forming physical barrier to prevent the spread of microorganisms. They also initially discovered that NETs may have harmful effects on the host and thereby stimulate autoimmunity, which opens a new field of neutrophil biology [2]. In the early stage, the process of NET formation is called NETosis [16]. In the latest expert review, it was emphasized that NETosis could not include all forms of NETs release, and it was recommended to avoid the use of the term "NETosis" or only in cases where neutrophil death was apparent, preferably using NETs formation [17]. Like many host protection mechanisms, NETs may also be a double-edged sword that can promote or prolong innate and acquired immune responses to a variety of diseases [6, 18]. Plenty of stimulation, such as physical and chemical stimulation [19, 20], inflammatory cytokines such as C5a and IL-8 [21, 22], and various pathogenic microorganisms and their derivatives [23, 24], determine the different mechanisms of the formation of NETs.

### The classical pathway of NET formation requires ROS

PMA stimulated neutrophils produce NADPH oxidase-2 (NOX-2)- dependent reactive ROS which lets neutrophils release NE referring to the nucleus, where it partially degrades specific histones and MPO, driving chromatin depolymerization independent of its enzyme activity [25–27], causing nuclear DNA moving and releasing out of the cell, and forming a reticular structure [27–29]. It has also been reported that some special stimulation (including immune complexes) touched neutrophils, mitochondrial ROS (rather than NOX-2-derived ROS) can drive similar NETs formation with the assistance of Ca2+ [20, 30, 31]. ROS, whether mediated by NOX or mitochondria, seems to be essential for the formation of NETs. Those NET formation takes a long time, usually lasts for several hours or even exceeds its lifespan and continues to resist the invasion of bacteria. When it causes neutrophils death, it is called "suicidal NETosis" [16]. Interestingly, when neutrophils are stimulated by GM-CSF, C5a or LPS, mitochondrial DNA (mtDNA), not nuclear DNA is ejected out of the cell to form NETs under ROS-dependent condition [32]. In this process, the lifespan of neutrophils is not affected. These results give neutrophil mitochondria a new role not only as a ROS generator, but also as a provider of DNA in the process of NET formation. In addition, recent studies have shown that optic atrophy1 (OPA1), a mitochondrial inner membrane protein, is essential to the process of NET formation, and its deficiency causes dysfunction in the release of DNA from the nucleus of human neutrophils, thus unable to form NETs [33].

### Pathway of ROS-independent NET formation

Although many studies have shown that PMA stimulation can easily form ROS-dependent lytic NETs, the mice model of skin infection with *Staphylococcus aureus* or *Candida albicans* shows that NET formation can be rapidly formed independent of ROS [34, 35]. In the process of the NET formation, NE translocation to the nucleus and chromatin depolymerization occur without obvious rupture of the nuclear membrane. The protein-modified chromatin was packaged into vesicles merging with the plasma membrane and nuclear DNA was released outside the cell through the vesicle transport mechanism without destroying the plasma membrane [18, 36]. Even though how these vesicles were released is not clear, it is clear that NETs formation leads to neutrophils maintain plasma membrane integrity without affecting their lifespan, and have the ability to move, chemotaxis, and phagocytize pathogens [37]. The process without ROS or NADPH oxidase is uniquely rapid, lasting about 5 to 60 min and requires strict supervision, which was triggered by toll-like receptors (TLR)2 or C3 complements. Importantly, NETs released without neutrophil death maintain their normal function [35, 38]. Recent studies suggest that there is a new mechanism of NETs formation independent of ROS, that is, bacterial toxins induce pores on the membrane of host neutrophils [39, 40]. As we all know, the NET formation is a process independent of caspase-3. Interestingly, it has been reported that NE and caspase-11 can process gasdermin D so that its pore-forming N-terminal forms pores in the nuclear and granular membranes as well as the plasma membrane. As mentioned above, it will promote NE migrate to the nucleus with the help of gasdermin D on the nuclear membrane, which may be due to the induction of NETs by calcium channel activation [41, 42].

### Signaling pathway in NET formation

Several different signaling pathways have been reported to play functional roles in NET formation. The first of these pathways is that PMA, LPS and various bacteria stimulation activate protein kinase C (PKC) through TLR and G protein related receptor (GPCRs), and then phosphorylate Raf kinase and activate Raf-MEK-ERK pathway [29, 43]. ERK phosphorylation NADPH oxidase complex and leads to the production of ROS. At the same time, neutrophils release NE destroying F-actin and MPO. ROS acts as a secondary messenger and promotes the transfer of NE and MPO from cytoplasmic granules to the nucleus, which catalyzing the interpretation of histone and leading to nuclear chromatin depolymerization [28, 44]. During this period, it is not clear how NE translates to the nucleus. Some studies have shown that nuclear membrane disintegration mediated by CDK4/6 may play an important role in NE nuclear translocation [45]. When neutrophils were activated, Ca2 + was released into the cytoplasm in response to the receptor agonist activating phospholipase C (PLC). PLC hydrolyzes phosphatidylinositol 4-diphosphate (PIP2) on the cell membrane to produce second messenger inositol triphosphate (IP3) and diacylglycerol (DAG), which contributes to the activation of intracellular Ca2+ and PKC respectively [46]. In fact, Ca2+ -assisted peptidylarginine deiminase 4 (PAD4) in the cytoplasm converts the positive charged arginine into neutral citrulline in the histone, destroying the electrostatic interaction of DNA- histones, weakening the skeleton structure and stability of chromatin, and finally leading to chromatin condensation [47]. In short, nuclear chromatin condensation can be achieved not only via post-translational modification of histone, namely PAD4-mediated histone citrullination [48] or acetylation [44, 49], but also NE-mediated histone hydrolysis [27, 29, 50].

Next, the rupture of the nuclear membrane is essential for the removal of nuclear chromatin out of the cell, and is also related to the source of NETs DNA. Amulic et al. reported the involvement of nuclear lamin A/C in NET formation [45]. They found that cyclin-dependent kinase 4 and 6 (CDK4/6) can control NET formation by regulating lamellar disintegration and rupture of nuclear membrane caused by phosphorylation of laminin A/C. In addition, some microscopic analyses showed that nuclear lamin B was also involved in the formation of NETs [51, 52]. The phosphorylation and dissociation of lamin B mediated by protein kinase C alpha (PKC α) is the reason for the disintegration / rupture of nuclear envelope. Lamin B is extruded from the ruptured nuclear membrane with depolymerized nuclear chromatin and can be decorated on the surface of extracellular NETs [52]. Interestingly, chromatin condensation and nuclear swelling are the main physical forces driving nuclear membrane rupture [53], which may provide a molecular basis for lamin kinase-mediated decomposition of the nuclear layer into the disintegration of nuclear membrane protein networks. Nuclear expansion forms a physical force from the inside out, which drives the disintegration of the nuclear membrane to expand until the whole nuclear membrane breaks, squeezing out the depolymerized nuclear chromatin. Finally, neutrophils release DNA outside the cell, and various proteins with bactericidal activity are connected to the DNA skeleton to form NETs.

## NETs in intestinal infection

There are many species of bacteria, fungi and several protozoan parasites associated with the induction of NET formation in the human intestinal tract [28, 54]. There is no doubt that the defense function of NETs against the invasion of pathogenic microorganisms [2]. However, Crane et al*.* proposed that in the model of bacterial enteritis, NETs help enteropathogenic *Escherichia coli* (EPEC)and Shiga-like *Escherichia coli* (STEC)attach to the intestinal mucosa by enhancing the biofilm function of microorganisms [55]. These findings hint that NETs play a dual role in intestinal infections. However, its mechanism has not been fully elucidated. Table 1 summarizes the production or changes of NETs when tissues or cells are infected by different microorganisms and the effects of their main components on tissues or cells. More studies are needed to elucidate whether the changes in NETs during infection are due to the infection itself or to experimental manipulation.

**Table 1:** Summary of the included studies of NETs in intestinal infection

Pathogen	Type of trial	The change of NETs	Active components of NETs	Treatment	The role of NETs and its effect to issues changes or cell	Mechanisms of antibacterial or non-antibacteri of NETs	Frist author, References
*Gram-positive* bacteria (staphylococcus)	Vivo, vitro	NET formed rapidly and NETs are released from the nucleus	NETs DNA	DNase I	NETs are crucial to contain an acute invasive infection in vivo	NET formation is tightly regulated and requires both Tlr2 and C3	Bryan et al. [35]
Enteropathogenic and Shiga-Toxigenic *Escherichia coli*(EPEC and STEC)	Vivo	Firstly reported the formation of DNA NETs in vivo in the intestinal tract. EPEC and STEC infections stimulate the formation of extracellular DNA NETs alonely	NETs DNA	DNase I	NETs were acting as an non-antibacterial host defense. Additing DNase provided protection against the intestinal tissue damage caused by EPEC infection		Crane et al. [55]
methicillin-resistant *S. aureus*(MRSA)	Vivo(WT mice,NE^−/−^ and PAD4^−/−^mice	Intravenous infection with MRSA, leading to rapid a neutrophil-dependent NET formation within the liver sinusoids	ExtDNA、NE and histones	DNase I	Neutrophil recruitment and subsequent NET release destroyed the tight junction between endothelial cells and is associated with profound liver injury	But the effectiveness of DNase might be limited in terms of removal of the most dangerous NET components and advocates for inhibition of NET production	Elzbieta et al. [56]
Afa/Dr Diffusely Adhering *Escherichia coli*	Vitro	NET production by PLB-985 cells infected with the Afa/Dr wild-type (WT) E. coli strain C1845	NET-bound proteases	DNase I	NETs is actively involved in the antibacterial response of PLB-985 cells against enterovirulent WT C1845 bacteria. But PLB-985-derived NETs might directly contribute to Caco-2/TC7 epithelial cell damage via NET-bound proteases		Marin-Esteban et al. [57]
*Pseudomonas aeruginosa*	Vivo and vitro	*P. aeruginosa* induces the production of NETs in vitro and in vivo and quickly responds and defends against the DNA and histone mediated-antibacterial effects of NETs by stabilizing the outer membrane	ExtDNA	DNase I, Mg^2+^ and Ptase	DNA backbone of NETs contributes to their bactericidal function. But the spermidine and *arn* surface modifications contribute to resisting a broad range of antimicrobial components present within NETs, which may protect *P. aeruginosa* from NET-induced oxidative damage	Produced new immune escape mechanisms by sensing and defending against NETs	Halverson et al. [58]
Nonpathogenic WT *Escherichia coli and* its several isogenic mutant	Vivo(*Lcn KO,mpoko* and *Nox2KO mice*) and vitro	Ent-producing *Escherichia coli* can NETs. Lcn2-deficient BMDNs generated more PMA-induced NETs than did WT BMDNs			The inhibition of neutrophil ROS and NET responses and impairs neutrophil function by enterobactin may confer a survival advantage to Ent-producing *Escherichia coli*	Showed the production of siderophore by E. coli and other bacteria may be a key mechanism that allows them to evade NET-mediated killing	Saha et al. [59]
*Vibrio cholerae*	Vivo and vitro	*V. cholerae* induces NET formation and degrades NETs by the activity of two extracellular nucleases Dns and Xds	ExtDNA		Dns and Xds mediate evasion of *V. cholerae* from NETs and lower the susceptibility for extracellular killing in the presence of NETs and enhanced survival of *V. cholerae* in the presence of NETs, leading to intestinal inflammation	Ecluded a new evidence that the innate immune response impacts the colonization of V. cholerae	Seper et al. [61]
*Entamoeba histolytica* trophozoites	Vitro	Neutrophils that were interacted with *E. histolytica* trophozoites released NETs. And the presence of both nuclear and mtDNA was detected. NETs were generated independently of NOX2-derived ROS	Histone H4, MPO, NE and decondensed DNA	DNase I, GSK484 and PMSF	NETs caught, immobilized and fragmented *E. histolytica* trophozoites	NETosis occurs rapidly and depends on the viability of amoebas. But mechanism the NETs formation triggered by this parasite and its role in protection or pathogenesis of amoebiasis not yet clarified	Ventura-Juarez et al. [65] DíAZ-GODíNEZ et al. [66]
*Candida albicans*	Vivo and vitro	Both opsonized and unopsonized *C. albicans* induce NET formation. NET formation in peritoneal cavity after *C. albicans* infection	NE	BB-CI-Amidine and GSK484	Neutrophil killing of unopsonized *C. albicans* requires dectin-2-mediated NET formation and the NETs restrains *C. albican*s spread from peritoneal cavity to kidney	Unopsonized *C. albicans*-induced NET formation is independent of NADPH oxidase and mitochondrial ROS and is dependent on PAD4 and dectin-2 enzymatic activity	Wu et al. [34] Branzk et al. [68]
*Aspergillus fumigatus*	Vivo and vitro	Reutrophils released NETs in response to *A. fumigatus* hyphae or large aggregated *A. fumigatus* conidia but failed to form NETs in response to small single conidia	NE	AREG (inhibitor of NE)	NETs not only were irrelevant in protecting these mice against the yeast-locked *hgc1*Δ*C. albicans* strain but also were detrimental to the host when present in large amounts	Selective NETosis was independent of the expression of molecules on the surface of fungi or the enzymatic activity of fungi and was regulated only by differences in microbe size	Branzk et al. [68]

### NETs and bacterial clearance

NETs have the proteolytic activity of NE. In infectious inflammation, such as methicillin-resistant *Staphylococcus aureus* (MRSA) infection, it has been observed that NETs can destroy the tight junction between endothelial cells and increase vascular permeability [56]. Moreover, this experiment shows that the absence of essentia MRSA toxins still caused NETs production and the consequent liver damage, which reminds us that in the future treatment of infectious diseases should not only remove bacterial toxins but prevent NETs formation in order to completely alleviate infectious injury. Diffusely adherent *Escherichia coli* expressing Afa/Dr fimbriae strain C1845 could also induce NETs to damage the F-actin cytoskeleton of human enterocyte-like cells and destroy the intestinal epithelium and that this deleterious effect is prevented by inhibition of protease release [57]. In order to avoid the capture of NETs, bacteria also adapt to other escape mechanisms. For example, *Pseudomonas aeruginosa* is more resistant to NETs than *Staphylococcus aureus* or *Escherichia coli*, resulting in less NET formation. There are several factors. Firstly, *P. aeruginosa* is the production of a microbial secreted DNase that degrade NETs DNA, which in turn restrict NETosis through non-representational mechanisms. Alternatively, DNA can induce expression of the *arn* or spermidine synthesis genes in *P. aeruginosa*, which in turn protect *P. aeruginosa* from NET-induced oxidative damage [58]. It suggests that *P. aeruginosa* can produce new immune escape mechanisms by sensing and defending against NETs. *Escherichia coli* also inhibit neutrophils producing ROS, form NETs by producing enterobactin (Ent), which is a catecholamine iron carrier used to isolate intracellular iron and unstable iron pools in neutrophils [59], which means the production of siderophore by *E. coli* and other bacteria may be a key mechanism that allows them to evade NET-mediated killing. In addition, the culture of intestinal bacteria in mice infected with *Citrobacter rotarius* showed that different bacterial subsets also had the ability to mobilize neutrophil oxidative outbreaks and initiate NET formation [60]. *Staphylococcus aureus* induces NETs by recruiting neutrophils during sepsis, and these NETs bind firmly to the hepatic sinusoids through histone-vWF interactions [35]. In 2013, the interaction between *Vibrio cholerae* and NETs was reported for the first time. It indicated that *Vibrio cholerae* can induce NET formation when it comes into contact with neutrophils. In turn, *Vibrio cholerae* secretes two extracellular nucleases Dns and Xds to rapidly degrade the DNA component of NETs in order to avoid and adapt to the presence of NETs. In other words, Dns and Xds mediate the escape of *Vibrio cholerae* from NETs and reduce the extracellular activity of NETs [61].

### NETs and intestinal amebae and fungal infection

Neutrophils are important host effector cells against amebae lysis parasites (*Echinococcus histolytica* trophozoites) [62, 63]. It was demonstrated that neutrophils activated by TNF-a and IFN-c were able to kill *E. histolytica* trophozoites, with MPO binding to the trophozoite plasma membrane and killing these invaders [64]. The part that NETs play in this process is not yet known. In 2016, Ventura-Juarez et al*.* for the first time identified that in vitro*,* while in direct encounter with *Echinococcus histolytica* trophozoites, neutrophils lost their circular morphology and integrity and variable length NET formation was observed which trapped, immobilized and fragmented *E. lysis* trophozoites. After neutrophils were pretreated with deoxyribonuclease I (DNase I), despite the fact that the nuclei of neutrophils contained histones, MPO and concentrated chromatin, they did not release NETs and the *E. histolytica* trophozoites did not show any damage, which indicates that released NETs from neutrophils have amebae killing effect [65]. In addition, it was demonstrated quantitatively that neutrophils treated with amebae trophozoites not only rapidly form NETs but also emerge with the simultaneous presence of nuclear and mtDNA. It is of interest that the formation of NETs was also found to be dependent on amebae activity, as heat-inactivated or paraformaldehyde-fixed amebae failed to induce NETs and, more interestingly, no ROS production was detected during neutrophil-amebae interaction, implying that amebae -induced NETs production is non-ROS-dependent [66].

The action of neutrophils on fungi bears a strong resemblance to that of amebae, using NETs to capture and destroy mycelium that cannot be engulfed by phagocytes [67]. *Candida albicans* hyphae have the capacity to trigger the formation of NETs, which are then trapped and killed by NETs, whereby the antifungal activity of NETs is mediated by calprotectin [68, 69]. Similarly, *Aspergillus* fumigatus mycelium can provoke the formation of NETs, which trap and inhibit fungal growth, possibly due to deprivation of the essential nutrient Zn2 + required by the fungus. However, in this case, NETs are not sufficient to kill Aspergillus fumigatus, as the NETs-mediated growth inhibition is eliminated by the addition of Zn2 + [70]. *Candida albicans* were NADPH oxidase-dependent. However, in a model of Candida albicans peritonitis, Wu et al*.* found a pathway for NETs independent of NADPH oxidase and similar to the chemically activated pathway, and they also discovered that Dectin-2-mediated PAD4-dependent NET formation in vivo prevented the spread of *Candida albicans* from the peritoneal cavity to the kidney [34, 71]. Nevertheless, PAD4 also seems not always to be required for NETs formation, and Guiducci et al*.* found that PAD4 is not required for antifungal immunity in mucosal and systemic *Candida albicans* infections, despite the fact that *Candida albicans* readily induces PAD4-dependent histone citrullination of neutrophils [72]. While most research on NETs and fungal-associated diseases has focused on *candidiasis* and *fumonis*, recent studies have illustrated that NETs also act in other fungal infections. For example, NETs can be seen in corneal scrapings from patients with fungal keratitis, a vision-threatening infection caused by a variety of fungi, including *Aspergillus*, *Fusarium*, *Candida* and *Streptomyces* [73]. The same applies in the case of *A. fumigatus conidia* infection [72]. In addition, it was recently reported that in a model of DSS-induced leaky gut lupus, intestinal fungi boosted the production of NETs, causing intestinal translocation of organic molecules and synergistically exacerbating the activity of lupus [73].

Large pathogens such as fungi and amebae activate the release of NETs from neutrophils in a similar signaling pathway, while at the same time the pathway of NETs release varies depending on the infecting pathogen, which may be relevant for clinical cure. Yet, more mechanisms of NETs release in response to amoebic and fungal infections are poorly understood and need to be further explored in the future.

## NETs and intestinal inflammation

Until now, numerous studies have documented that the expression of NETs is increased in inflamed intestinal mucosa, feces or blood, and that NETs abundance positively correlates with the degree of inflammatory intestinal disease, and that destruction of NETs by DNase I ameliorates the systemic inflammatory response, intestinal epithelial cell apoptosis and intestinal injury [74]. Table 2 summarizes studies and therapeutics used to target NETs in intestinal inflammation.

**Table 2:** Summary studies and therapeutics used to target NETs in intestinal inflammation

Model/Disease	The change of NETs	Therapeutic Agents	The role of NETs and its effect to issues changes or cell	Mechanisms of pro-inflammatory or Anti-inflammatory of NETs	Author, References
Lethal endotoxemia, vivo, vitro	NETs are formed in lungs during the lethal endotoxemia and that inhibition of PAD4 can abolish NET formation	YW3–56(PAD2/PAD4 inhibitor)	NETs can cause leakage of endothelial cells and that different NETs induce permeability to different extents and YW3–56 alleviates LPS-induced ALI, educing organ damage and raised survival rates	PAD-NET-CitH3 pathway	Liang et al. [77]
Endotoxaemia, vivo	Avast distribution of NETs was observed in the liver vasculature of endotoxemic mice. Visualization of the terminal product of coagulation, fibrin, demonstrated colocalization with NETs	DNase I	NETs promote intravascular coagulation during sepsis. NET-induced intravascular coagulation is a fundamental contributor to microvascular hypoperfusion in sepsis. NETs are key pathological mediators in systemic intravascular coagulation and subsequent end-organ damage in bacterial sepsis	A dynamic NET–platelet–thrombin axis that promotes intravascular coagulation and microvascular dysfunction in sepsis	Mcdonald et al. [75]
CLP, vivo, vitro	Induction of sepsis caused significant formation of NETs	rhDNAse	NET formation exerts proin-flammatory effects in septic lung injury	NET activity regulates CXC, TNF-α, and HMGB1 chemokine formation	Luo et al. [76]
Lethal septic shock, vivo, vitro	NETs were significantly elevated in abdominal sepsis patients and there were significant correlations between NETs markers and intestinal damage markers in serum	ODN2088(TLR9 antagonist)	NETs participate in sepsis-induced intestinal apoptosis. NETs impair the integrity of intestinal epithelial cell monolayer barriers in vitro	TLR9–ER stress signaling pathway possible participate in NETs-induced intestinal epithelial cell death	Sun et al. [78]
trauma hemorrhagic shock, vivo	Trauma-hemorrhagic shock induced the formation of intestinal NETs and disrupted the intestinal tight junction proteins	DNase I	NETs contribute to the dysfunction of the intestinal barrier in T/HS and aggregated intestinal injury	Tranexamic acid appears to suppress NETs formation via the classic ROS/MAPK pathway	Chu et al. [79]
ICU patients, Ex vivo	NETs can be directly induced by incubating neutrophils with plasma or sera from patients with sepsis	Inhibition of IL-8 or MAPK	Sepsis is the predominant ICU condition associated with NET formation and degrees of NET formation predict DIC development and are associated with multisystem organ failure and mortality	MAPK activation as the major pathway of IL-8–induced NET formation in patients	Abrams et al. [87]
Midgut volvulus, vivo	A significant increase in ceDNA within 4 h post midgut volvulus	DNase1 and tPA / LMWH	Formation NETs disrupts intestinal tissue integrity after torsion	Diminished the inflammatory response	Boettcher et al. [91]
Intestinal I/R, vivo	NETs are present in the intestine and that cfDNA is released into the blood during intestinal I/R injury	DNase-1	NETs contribute to the early inflammatory response after intestinal I/R injury	Dnase could reduce NET density, downregulate the proinflammatory response, changes and maintain the functional integrity of tight junctions and the cytoskeleton	Wang et al. [92]
Intestinal I/R, vivo, vitro	Pre-ischemia, the number of NETing leukocytes was modest and postischemia NETosis was vastly enhanced in vivo. Isolated bone marrow-derived neutrophils from germ-free mice and broad-spectrum antibiotic-treated mice show hyperreactive LPS-induced NETosis	Broad-spectrum antibiotics	NETs mediated mesenteric I/R injury	Tonic stimulation of the cell-intrinsic TLR4/TRIF (TIR-domain–containing adapter-inducing interferon-β) signaling pathway by gut commensals attenuates LPS-induced NETosis	Ascher et al. [93]
NEC, vivo, Ex vivo	NETs presented in human NEC ileum.NET formation was abundant in the small intestine of pups in the NEC group	Cl-amidine	NETs are critical in the innate immune defence during NEC in preventing systemic bacteraemia. NETs inhibition increases inflammation, bacteraemia and mortality in murine necrotizing enterocolitis	NET formation may be disease- and model-specific, and in NEC, they depend largely upon the level of intestinal bacterial translocation	Chaaban et al. [11]
NEC, vivo	NEC significantly induced elevated cfDNA	DNase1	NETs induced tissue damage, oxidative stress, and inflammation	DNase1 treatment significantly reduced TLR4 and C5a receptor expression and thus interfered with the typical inflammatory cascade	Klinke et al. [99]

### NETs and sepsis

In the development of sepsis, neutrophils migrate from circulating blood to infected tissues and mediate the formation of NETs, which kill pathogens. However, NETs component histones and NEs are as well toxic to host epithelial and endothelial cells. In an animal model of bacterial sepsis, DNase administration reduced organ damage and raised survival rates [35], and yet, DNase is administered prophylactically, which is impractical to patient care, and to further test this conclusion, the use of PAD4-/- mice to prevent the forming of NETs has shown to be protective against LPS-induced endotoxaemia, which indicates that NETs do exacerbate damage in sepsis [75]. Intriguingly, recombinant human DNase I administration in a model of sepsis with cecum ligation and perforation (CLP) depleted NETs, impeded early immune responses in mice and delayed bacterial clearance, thereby exacerbating pathological changes in lungs and liver [76]. This serves as a reminder that further studies are needed to document the utility of PAD4 and DNase I in sepsis. Septic patients frequently present with intestinal dysfunction and lesions, and neutrophils infiltrate and release NETs in the intestine of LPS-induced endotoxaemic rats. In sepsis, lipopolysaccharide (LPS) induces PAD4 activation and NET formation via the PAD-NETs-CitH3 pathway, leading to altered permeability of pulmonary vascular endothelial cells [77]. NETs contribute to sepsis-induced intestinal barrier dysfunction through modulation of the TLR9-mediated endoplasmic reticulum (ER) stress pathway, which encourages inflammation and apoptosis, and suppression of the TLR9-ER stress signaling pathway attenuates NETs-induced intestinal epithelial cell death [78]. Indeed, traumatic hemorrhagic shock can also instigate the NETs formation in the intestine, disrupting intestinal tight junction proteins, and the clearance of NETs by DNase I mitigates intestinal injury [79].

Excessive activation of neutrophils, however, can facilitate the formation of immune thrombi and even provoke disseminated intravascular coagulation (DIC), which can impair the microcirculation. A *vivo* imaging study revealed that the NETs-platelet-thrombin axis fosters intravascular coagulation in the liver during endotoxaemia [75] and that Poly P, a potent activator of thrombin, and NETs work together to promote DIC [80], which would explain why DNase or PAD4 deficiency inhibits net formation to reduce tissue damage, and blocking NETs and inhibiting intravascular coagulation potentially ameliorates organ reperfusion and attenuates organ damage. Certainly, NETs trigger pro-thrombotic and pro-coagulant platelet-mediated responses through interactions with TLR4, and LPS activation of platelets could elicit platelet-dependent tissue factor procoagulant activity (TF-PCA) and boost thrombin production in a TLR4-dependent manner, and as a critical receptor, TLR4 on platelets is likely to be an influential element in septic DIC [81]. Increased P-selectin expression and increased platelet-neutrophil and monocyte aggregation have also been found in COVID-19 patients, which is associated with thrombotic complications [82]. Vascular endothelial cells have a significant effect in sepsis thrombosis and NETs magnify the endothelial dysfunction associated with thrombosis [83, 84]. Thus, the interaction of NETs with platelets, complement and endothelium mediates, to some extent, the sepsis immune thrombosis.

Actually, NETs have become a therapeutic target in critical diseases [85], and real-time monitoring of the extent of NETs potentially could be beneficial in clinical practice for critically ill patients. Hu et al*.* observed increased formation of the NET structure and elevated expressions of NET-associated proteins in intestines of critically ill surgical patients and early enteral nutrition preserved intestinal barrier function through reducing the formation of NETs in critically surgical patients [86]. Using a novel assay in a prospective cohort study of 341 ICU patients and identified a significant correlation of NETs formation with disease severity, that is, robust NETs formation was found in sepsis and independently predicted the occurrence of DIC and mortality. They also confirmed that IL-8 is the main factor driving NETs formation through the MAPK pathway, and inhibition of IL-8 or MAPK can significantly reduce NETs formation. Therefore, this test can provide information about NETs forming ability and its inducing factors in vivo, thus guiding clinical management, identifying patients' targeted therapy and personalized NETs inducing factors, and then improving the treatment targeting strategy of ICU patients [87].

The outcome of sepsis depends on early understanding and intervention, so the clinical evaluation of NETs function may be a valuable biomarker for early diagnosis of sepsis.

### NETs and intestinal ischemia reperfusion injury

Intestinal ischemia reperfusion (IR) injury is a phenomenon in which intestinal injury is aggravated by restoration of blood flow based on intestinal ischemia from various causes, and even irreversible injury occurs, often after shock, trauma, acute intestinal ischemia and intestinal transplantation [88]. Following clinical occurrence of intestinal I/R injury, it often gives rise to intestinal bacterial translocation, endotoxin emigration, and massive release of inflammatory cytokines leading to liver, kidney, lung, and other multi-organ damage, which in turn causes systemic inflammatory response, systemic multi-organ dysfunction syndrome, multi-organ and tissue failure, and even death [89]. For many years, neutrophils have been the leading cause of inflammation caused by IR injury. For years, neutrophils have been responsible for the inflammation caused by IRI. During mesenteric infarction, neutrophil recruitment determines the extent of IR injury.

The first study regarding ischemia–reperfusion and the role of NETs, Oklu et al*.* established an IR model on the unilateral hind limb of mice and demonstrated the possible involvement of NETs in myofiber injury [90]. Inspiration from this finding inspired later investigators to focus on IR in other organs. Boettcher et al*.* demonstrated firstly in an experimental model of intestinal IR injury that neutrophils released extracellular DNA in the form of NETs, which were involved in organ damage, and DNase1 treatment diminished the inflammatory response, including NETs, without increasing the risk of bleeding, while extracellular DNA-targeted therapy also improved the regression of intestinal IR injury in neonatal rats [91]. Above results imply that targeting extracellular DNA possibly provides a safe therapeutic approach for future patients with intestinal infarction. Wang et al*.* further evaluated the therapeutic value of DNase-1 in a rat model of intestinal IR injury based on exploring whether NETs engage in the pathogenesis of intestinal IR injury. The results indicated that extracellular DNA was readily detected in rat serum after 1 h of ischemia and 2 h of reperfusion, and that DNase-1 treatment obviously attenuated the inflammatory response, restored intestinal barrier integrity, and enhanced the expression of tight junction proteins [92], which offered further evidence that DNase-1 has the potential to be an effective treatment for attenuating intestinal IR injury.

Recently, an increasing number of reports have proved that the gut commensal microbiota serves an essential role in mitigating organismal injury in acute mesenteric ischemia, but whether the specific mechanisms are related to NETs has been incompletely elucidated. To investigate the impact of intestinal microbiota in acute mesenteric infarction, Ascher et al*.* found in a mouse IR model that neutrophils with more recruitment and higher reactivity and markedly increased NETs were found in conventionally reared mice treated with antibiotics or in germ-free mice, whereas in conventionally reared mice or mice colonized with minimal microflora altered by *Schaedler's flora*, NETs were attenuated by a mechanism possibly related to activation of the TLR4 /TRIF (TIR-domain-containing adapter inducing interferon-β) signaling pathway, implying that the gut microbiota inhibits the high reactivity of mesenteric I/R-injured neutrophils, reduces the NETs, and is protective against IR in mice [93]. Nonetheless, knowing that short chain fatty acids (SCFAs) are mainly produced by bacterial fermentation of dietary fiber and is a catabolic product of the intestinal flora. Research has found that SCFAs stimulate the formation of NETs in vitro and that the result was probably mediated partly by the free fatty acid 2 receptor (FFA2R) expressed in neutrophils [94]. Similarly, Li et al*.* evaluated the role of microbiota metabolite butyrate in modulating NETs in IBD in a mouse DSS model and found that butyrate improved mucosal inflammation by ameliorating neutrophil-associated immune responses including inhibition of NETs [95].

Regarding the additional link between the formation of NETs and gut microbes, a study by Liang et al. in 2019 revealed that oral administration of Staphylococcal nuclease (SNase), a nuclease that degrades DNA or RNA, was capable of effectively degrading NETs in vitro and in vivo, thereby improving intestinal barrier function. More importantly, SNase alleviated the intestinal inflammatory microenvironment and averted the development of type 1 diabetes (T1D) in non-obese diabetic (NOD) mice by altering the species richness and composition of the intestinal microbiota of NOD mice [96]. Recently, a study has also shown that dominant microbes (*Acetatifactor, Coprococcus2, Lachnoclostridium_5 and Lachnospiraceae_FCS020_group*) in NOD mice had a positive correlations with neutrophils and could possibly affect T1D via NETs [97], which inspires us that further works concerning the specific mechanisms of the interaction between intestinal microbiota and NETs require more studies to be revealed.

Apart from local injury, intestinal IR injury can also harm other distant compartment organs, including the liver, and the process mainly occurs via histone, network formation and cytokine storm induction [11]. Hayase et al*.* discovered that accumulation of histones and NETs was found in the liver and intestine after intestinal IR occurred in mice, as well as that process exacerbated distant liver injury. Recombinant thrombomodulin attenuated the liver injury caused by IR by inhibiting the accumulation of histones and NETs in the liver [98].

### NETS and necrotizing enterocolitis

Necrotizing enterocolitis (NEC) is a devastating gastrointestinal disease affecting preterm infants. Characterized by intestinal inflammation and leukocyte infiltration, which often progresses to necrosis, perforation, and, in severe cases, death [99]. Neutrophils, the first-line responders of the neonatal innate immune system against infection, can eliminate pathogens through phagocytosis, degranulation, and formation of NETs. However, it is widely known that excessive NET formation or delayed clearance of NET components (especially histones) causes pathological conditions such as sepsis, thrombosis and transfusion-related acute lung injury [75, 87, 100]. Therefore, whether NETs offer protection against abnormal intestinal pathogens or contribute to pathology in models of NEC that require intestinal infection is still unclear.

Chaaban et al*.* found that circulating nucleosomes are present in premature human infants with NEC and presence of neutrophil extracellular traps in human NEC ileum. Notably, Cl-amidine treatment almost doubled the mortality of the pups exposed to dithizone/Klebsiella (DK)-induced NEC mice model, and intestinal mucosal tissue sections showed moderate to severe damages. In addition, IL-1β levels, blood BUN, creatinine and ALT were remarkably elevated in NEC + Cl-amidine compared with NEC pups, suggesting that inhibition of NETs appears to further exacerbate systemic inflammation and organ damage in mouse NEC and is likely due to increased bacteremia [101]. Surprisingly, in a study by Vincent et al. yielded seemingly contradictory findings by establishing a NEC mouse model different from DK-NEC model established by Chaaban et al. They found that serum circulation free DNA (cfDNA) was positively correlated with clinical manifestations of NEC, and markers of neutrophil activation and NETs were significantly increased in animals suffering from NEC and in human samples compared to controls [102]. Prevention of NET formation in mice by suppressing PAD significantly reduced NEC-induced mortality, tissue lesions and deterioration in mice. What's more interesting is that immunohistochemical results of mouse NEC model were positively correlated with the results of human NEC specimens, the NETs marker observed in mice could potentially be used to study the pathogenesis of human NEC [102]. Similar conclusions were reached in the study by Klinke et al*.* Furthermore, systemic DNase1 treatment dramatically lowered NEC severity and mortality, and the outcomes were confirmed in human subjects [103], Therefore, DNase1 is considered as a therapeutic option for NEC neonates.

Given that the above-mentioned inhibition of PAD4 formation appears differently in mouse NEC models, there are several factors as follows. Firstly, administering Cl-amidine in a different murine model of NEC leads multitude uncertainties in the formation of NETs (NETs generation time and how much to generate, etc.). That is, the puzzling effects exerted by NETs are disease- and model-specific. Secondly, Cl-amidine delivery at different times in the development of NEC also produces different impacts. More importantly, NETs as an immune response modality seems to represent a microcosm of physiological trade-offs. NEC-induced NETs may be vital in the early stages of disease to prevent bacteremia, but are detrimental in later periods when excessive inflammation accumulates leading to tissue destruction [101]. It helps guide us in the future study to design more rational, accurate and closer to the human condition disease models.

Considering the high incidence of NEC in children with congenital heart disease (CHD) after pharmacological or surgical intervention, Polin et al*.* proposed in 1976 to isolate cNEC from NEC [104]. Nowadays, for a finer understanding of NEC pathogenesis and better diagnosis and treatment, NEC is stratified into typical inflammatory NEC(iNEC) occuring mainly in preterm infants and cardiac NEC (cNEC). Children with CHD suffer from reduced cardiac contractility and inadequate blood oxygenation, causing inadequate blood supply to the superior mesenteric artery, contributing to reduced bowel liner perfusion and, when treated medically or surgically, blood flow to previously underperfused areas of the intestine, which leads to intestinal I/R injury [105]. Ultimately, intestinal I/R injury causes an excessive inflammatory response through activated neutrophils and abnormal intestinal flora, leading to the development of cNEC [106].

The ultimate outcome of NEC is intestinal inflammation, and it causes the release of cytokines that permit the migration of neutrophils to the site of inflammation. As described previously, NETs are involved in intestinal I/R injury and excessive intestinal inflammation [74, 92]. We understand that the incidence of cNEC is intimately linked to I/R damage, so does I/R injury triggered by NETs involved in the development of NEC? In the latest clinical retrospective analysis study comparing neonates with cNEC to those with iNEC, staining for NE and H3cit showed a significant increase for neonates diagnosed with cNEC in comparison to neonates with iNEC. And the concentration of neutrophils was substantially higher in the cNEC group. It can be hypothesized that NETs partially mediate the process of intestinal I/R injury in cNEC [12]. The role of neutrophils and NETs in the pathogenesis of NEC has not been fully elucidated, and more prospective studies are needed to verify and explore them as potential diagnostic parameters.

## NETs and Inflammatory Bowel Disease (IBD)

IBD is a chronic intestinal inflammatory disease that involves innate and acquired immune responses, mainly ulcerative colitis (UC) and Crohn's disease (CD), with a multifactorial pathogenesis involving genetic susceptibility, epithelial barrier defects, dysregulated immune response, and environmental factors [107, 108]. As a potential disease mechanism, NETs play an important role in a variety of immune-mediated diseases, including IBD, systemic lupus erythematosus, rheumatoid arthritis and so on [109–111].

### NETs and Ulcerative colitis (UC)

UC is a chronic nonspecific inflammation that repeatedly invades the colon and rectum. Early proteomic analysis of UC patients indicated that the abundance of calprotectin and lactotransferrin in colonic tissue associated with the level of inflammation, in addition to the microscopic observation of 11 proteins with elevated abundance in UC biopsy tissue linked to NETs [14, 112]. Later, investigators analyzed data on the protein and peptide levels using linear mixed-effects regression models and reached the same conclusions [113]. To further explain the relationship between NETs and UC, many investigators have invested in clinical and experimental studies of UC. Among them is a cohort study that assessed patients with UC and those without a diagnosis of IBD through colon biopsy. Western blot results revealed that NETs correlated with the expression of PAD4 in the intestinal mucosa. PAD4 spurred the release of NETs through citrullination histones, leading to chromatin decondensation and DNA release, and, NETs were located mainly in the mucosa of UC, suggesting that NETs release was related with characteristic anatomical damage [114]. A in vitro study analyzing the NETs-related proteins in colon tissues from patients with UC, CD and colon cancer demonstrated that PAD4, MPO, NE and citrullination histone H3 were highly expressed in pathological tissues of UC compared to CD, that UC-associated neutrophils yielded more NETs following TNF-α stimulation, and that those proteins expression was declined upon administration of anti-TNF-α therapy [115]. It suggests that a positive regulatory relationship exists between TNF-α and NETs in UC patients and TNF-α spurs the forming of NETs, and NETs in turn boosts the secretion of TNF-α. In another report, the researchers initially analyzed neutrophils in peripheral blood and colon tissue of 48 patients with IBD and showed that patients with active disease exhibited more NETs release than those with inactive lesions [116]. Likewise, Angelidou et al*.* studied that NETs production was higher in patients with active UC compared to patients with CD and healthy patients, Interestingly, more IL-1β and tissue factor thrombin (TF) were found in NETs obtained from colonic tissue and blood of these patients, and the production of these was linked to Redd1 protein-induced autophagy [117], which is interrelates NETs with programmed death and provides a direction for later studies on the mechanisms of NETs. Importantly, immunohistochemical analysis of PAD4 in UC patients by Abd EL Hafez A et al*.* revealed high expression of PAD4 in UC colon tissue compared to normal colon tissue, illustrating the prognostic and therapeutic value of NETs-related markers in the colon tissue of UC patients and guiding patients to targeted therapy with selective PAD4 inhibitors [114].

Circulating extracellular DNA (ceDNA) is widely known to worsen the prognosis of many diseases. CeDNA released by neutrophils during infection or inflammation is present as NETs. In an experimental mouse model of colitis, plasma total ceDNA concentrations with increasing inflammation were found to increase, and was accompanied by an increase in endoscopic colonic damage scores and the percentage of neutrophils forming NETs [118]. In another experimental study, dextran sodium sulfate (DSS)-induced abundant NETs in the colon of mice induced apoptosis of epithelial cells and disrupted tight junctions, compromising the permeability of the intestinal mucosal barrier and causing increased bacterial translocation and inflammation in the intestinal lumen [119]. The production of NETs enhances the production of TNF- α and IL-1β by activating the ERK1/2 signaling pathway. The degradation of NETs reduces colitis and prevents increased expression of pro-inflammatory factors as well as neoplasia and thrombosis relevant to IBD [115, 120]. In terms of thrombosis, NETs release phosphatidylserine (PS) by being prone to a prothrombotic state, and LPS activates TLR2 and TLR4 in platelets and endothelial cells, thereby inducing pro-coagulant properties and leading to thrombosis [116].

### NETs and Crohn's disease (CD)

CD is a chronic transmural inflammatory bowel disease of undetermined etiology, with symptoms invading the entire GI tract, but the most typical lesions are concentrated in the terminal ileum and its adjoining colonic terminal [121]. Studies on NETs in CD mostly focus on basic experimental studies. The use of 2,4,6-trinitrobenzene sulfonic acid (TNBS) to induce the establishment of a mouse CD model reveals augmented expression of Ly6G, citrullinated histone H3 (CitH3) and PAD4 in mouse colonic tissues and an enhanced ability of neutrophils to produce NETs in vitro [122]. Blocking the formation of NETs efficiently attenuates the clinical colitis index and tissue inflammatory response in TNBS mice and regulates the expression of pro- or anti-inflammatory cytokines. Consistent with the DSS-induced colitis model, damage to the intestinal mucosal barrier and apoptosis of epithelial cells were also seen in the TNBS-induced mouse model [112, 123]. Proteomic and metabolomic analyses of colonic tissues from CD patients indicated an upregulation of NE expression of metabolic proteins associated with NETs compared to healthy subjects and showed marked differences in metabolic protein abundance and calprotectin [124].

The above clinical and experimental findings indicate that in IBD, particularly UC, there is greater release of NETs, which causes higher damage to the colonic tissue, presents characteristic features of IBD disease, and predisposes patients to extra intestinal pathologies, such as thrombosis. The use of treatments targeting NETs components in IBD has been reported, where inhibitors targeting PAD4, elastase and NETs-related DNA reduce the clinical manifestations of these diseases. However, there is a need for further studies to evaluate this therapeutic strategy [111].

## NETs and intestinal cancer

A growing number of research has shown that tumor cells and tumor microenvironments stimulate neutrophils and induces the release of NETs from various cancer types [125–127]. Neutrophils are well-known mediators in tumor biology, but their role in solid tumors has been redefined by NETs. NETs have recently been detected in specimens from six different human solid tumors, including colorectal cancer (CRC), and they showed substantial individual differences in tissue density and distribution, and it was concluded that NETs were positively correlated with IL-8 and negatively correlated with tumor-infiltrating CD8 + lymphocytes [128]. Given that platelet-derived poly P drives the release of NETs from neutrophils, et al. used biopsies of adenomas, hyperplastic polyps, IBD and healthy colon tissue were as a control study and found that in CRC, CD68 + mast cells expressing Poly P are one of the factors that stimulate the release of NETs from neutrophils, and mast cells with detectable CD68 + poly P expression could represent a potential prognostic marker for colorectal adenoma and/or carcinoma [129].

With the growing number of more studies, the mechanisms related to NETs their actions on tumor tissues are slowly starting to be revealed, including direct effects to the cancer cells and changes in the tumor microenvironment, such as promotion of tumor growth [130], promotion of metastasis [131], awakening from a dormant state [19, 132], and promotion of escape of cytotoxic immune cells [133, 134]. Recent studies have indicated that NETs are involved in the entire invasion-metastasis cascade of tumors [135]. NE released from NETs promotes further acceleration of colorectal tumor growth by upregulating PGC-1 through activating TLR-4 in cancer cells and enhancing mitochondrial biosynthesis [136]. In LPS-injected CRC mice, cancer cells probably foster the NET formation by TLR9 and mitochondria-activated protein kinase signaling pathways, and the analysis of clinical data from CRC patients showed a striking relationship between the NET formation and the rate of metastasis and survival [137]. CRC cells may translocate mutated KRAS to neutrophils via exons, thereby boosting the NET formation by modifying IL-8 and ultimately leading to CRC aggravation [138].

In CRC models in mice, NETs are formed extensively and depletion or inhibition of NET formation can considerably lower the amount of tumor metastasis [135]. Feedback regulation between elevated IL-8 and NETs in CRC can promote liver metastasis in CRC [139], and NETs-associated CEACAM1 can also serve as a potential therapeutic target for the prevention of colon cancer metastasis [140]. NETs exert a major action in colon cancer intraperitoneal metastasis via regulation of colon cancer cell migration and adhesion to extracellular matrix proteins [138]. Using a clinical mouse model of colon cancer combined with in vivo video microscopy, Rayes et al*.* confirmed that NETs facilitate the adhesion of circulating tumor cells (CTCs) to the lung and liver, thus functionally contributing to metastatic progression, whereas blocking NET formation by multiple measures markedly inhibits spontaneous metastasis [125]. Low-density lipoproteins foster the retention of CTCs via NETs and suppress T cell-mediated antitumor responses in target organs, hence prompting postoperative tumor metastasis [141]. Ample evidence shows that certain cancer cells have a high organismal preference for colonization and metastasis to certain distant organs, with colon cancer cells more prone to metastasize to the liver and lung [142]. Liver metastases in patients with colon cancer are rich in NETs, which usually corresponds to the metastatic organ tropism of colorectal cancer [143]. This was most likely connected to the NET-DNA receptor-transmembrane protein CCDC25 on the surface of cancer cells, which senses extracellular DNA and thus initiates the ILK-β-parvin pathway to enhance the motility of cancer cells. As we know, DNase I alters the function of NETs by cleaving the DNA strand. Xia et al*.* established a mouse model of CRC liver metastasis using an adeno-associated virus (AAV) gene therapy vector that specifically expresses DNase I in the liver, which in turn proved that AAV-mediated DNase I gene transfer can be a safe and effective way to curb liver metastasis [144], hinting at new therapies for CRC.

Cancer-related thrombosis is strongly linked to poor prognosis, and patients with CRC are generally at higher risk of suffering from venous thrombosis, yet the exact mechanism remains unknown. It has been shown that platelets in CRC patients stimulate neutrophils to produce NETs, which can be inhibited by depletion of HMGB1, and that the level of NETs in the blood of CRC patients increases in parallel with cancer progression, leading to a shortened clotting time and a significant increase in thrombo-antithrombotic complexes and fibrin fibrils, compared to healthy subjects. Interestingly, when exposed to NETs from CRC patients, endothelial cells were also converted to a procoagulant phenotype. This finding reveals a complex interaction between neutrophils, platelets and endothelial cells [145]. As well, tumor development and hypercoagulation was also found to be related to neutrophils in a mouse model of small intestinal tumors [146]. Finding that intestinal tumorigenesis is associated with aggregation of low-density neutrophils, which have a pre-tumorigenic N2 phenotype and spontaneous NETs formation, and that elevated circulating lipopolysaccharide induces upregulation of complement C3a receptors on neutrophils and activation of the complement cascade, which consequently leads to NETs division, inducing coagulation and N2 polarization, thus promoting tumorigenesis. It lays the foundation for a new link between tumorigenic hypercoagulation, increased NETs and complement activation, thereby providing a favorable explanation for the promotion of tumor development by blood coagulation [147]. We therefore consider that NETs potentially offer new therapeutic targets for preventing the risk of thrombosis in patients with CRC.

In addition, high levels of NETs are linked to a poorer prognosis of cancer. High levels of NETs in the blood of patients with colorectal cancer were correlated with postoperative complications and tumor recurrence rates [135, 148, 149]. Patients with metastatic colorectal cancer have elevated NETs in tumor tissue, and greater preoperative serum MPO-dsDNA levels resulted in shorter survival time [136]. Richardson et al*.* identified a novel neutrophil phenotype in patients undergoing CRC resection, showing reduced forming of NETs, reduced apoptosis, and increased phagocytosis. In other words, the accumulation of neutrophils in the circulation as a result of damaged cell death may be of potential harm to the postoperative host and an early phenotypic switch may be desirable [150]. However, the role of NETs for tumors is not restricted to only promoting tumor growth and metastasis; Arelaki et al*.* obtained tumor tissue sections and metastatic lymph nodes from ten patients with colon adenocarcinoma and found that TF-bearing-NETs and neutrophil localization were evident, with a gradual decline in neutrophil infiltration and NETs concentration from the tumor center to the distal margins. Interestingly, NETs created in vitro impeded cancer cell growth by inducing apoptosis and/or inhibiting proliferation [151].

Above findings showed that NETs are available as biomarkers to guide clinical diagnosis and treatment, and to assess the prognosis of cancer patients, and NETs will emerge as a new target for treatment and intervention of intestinal cancers. Table 3 summarizes the major effect of the studies describing NETs in CRC.

**Table 3:** Summary the major effect of the studies describing NETs in CRC

Study design	Cell type	Potential mechanisms to induce NET formation	The role of NET in tumor progression	Frist author, References
In vivo and in vitro	Murine colorectal (MC38) cells, HCT116, Hepa1-6, and Huh7 cell lines	Increased citrullinated histones and circulating MPO-DNA levels were related to poor survival of CRC patients	NETs can directly alter the metabolic programming of cancer cells to increase tumor growth and shorter survival time	Yazdani et al. [129]
In vivo, in vitro nd ex vivo	Human colorectal cell line HCT116 or luciferase-labeled HCT116 cells	TLR9 and the mitogen-activated protein kinase signaling pathway	LPS-induced formation of NETs in promoting the development of tumors and metastasis	Wang et al. [130]
In vitro and Ex vivo	Human acute myeloid leukemia (AML) cells, Caco-2 cells	–	Confirmed presence of NETs within the primary tumor sites of CRC and gradually dispersed to the tumor boundary, particularly to nearby metastatic lymph nodes	Arelaki et al. [122]
In vivo and in vitro	DKs-8(WT allele) cells and DKO-1 (KRASmutant)cells	Exoxsomes from KRAS mutant CRC increase IL-8 production and provoke NET formation	Released NETs increase CRC cells growth	Shang et al. [131]
In vivo, in vitro,and Ex vivo	Human hepatocellular carcinoma, human cell line HT29, mice cell line MC38	Elevated tumorous interleukin (IL)-8 expression triggered by NETs and overproduced IL-8 in turn activate neutrophils towards NETs formation	Increased NETs boosted tumorous proliferation and invasion and contributed to onset of CRC liver metastasis	Yang et al. [132]
In vivo and in vitro	Human colon carcinoma cell line (HT-29), murine colon carcinoma subline with low CEACAM1 expression (MC38CC1-),murine colon carcinoma subline stably transfected with CEACAM1 long isoform[MC38CC1L]	NET-associated carcinoembryonic Ag cell adhesion molecule 1 (CEACAM1) as an essential element for this interaction	NETs can promote colon carcinoma cell adhesion, migration and metastasis	Rayes et al. [133]
In vivo and in vitro	Murine Lewis Lung carcinoma cell subline H59, Murine colon carcinoma cell line MC38	Primary colon cancer cells provoked NETs generation	Prime adhesion of CTCs to the liver and degradation of NETs decreased CRC cell adhesion and spontaneous metastasis to the liver and lung	Rayes et al. [118]
In vivo and Ex vivo	human colon cancer cell line HCT116,	The transmembrane protein CCDC25 as a NET-DNA receptor on cancer cells that senses extracellular DNA and subsequently activates the ILK-β-parvin pathway to enhance cell motility	A transmembrane DNA receptor that mediates NET-dependent metastasis	Yang et al. [136]
In vivo and in vitro	Human hepatoma cell line HepG2, murine colon carcinoma MC38	Neutrophil infiltration and NET formation reduced by adeno-associated virus (AAV) based DNase I gene therapy	Reduced liver metastasis	Xia et al. [137]
Ex vivo	Human umbilical vein endothelial cells (HUVECs)	platelets from CRC patients stimulated healthy neutrophils to extrude NETs, which could be inhibited by the depletion of HMGB1	NETs induce the procoagulant activity PCA and promote hypercoagulable state in CRC	Zhang et al. [142] Guglietta et al. [139]
In vivo, in vitro,and Ex vivo	MC38 and Luciferase-expressing MC38 cells (MC38/Luc)	NET triggered HMGB1 release and activated TLR9-dependent pathways	NETs further fuel cancer cells adhesion, proliferation, migration, and invasionthe and reduce more than fourfold disease free survival	Tohme et al. [128]
Ex Vitro	Systemic neutrophils were isolated from human		Adverse patient outcomes were associated with increased preoperative NETs production	Richardson et al. [142, 143]

## Conclusion and future direction

NETs, a double-edged sword in which neutrophils exert immunomodulatory effects, are involved in the occurrences of various diseases, especially intestinal diseases. On the one hand, the production of NETs by neutrophils prevents pathogenic microbial invasion and reduces intestinal damage caused by intestinal inflammation, and on the other hand, pharmacological inhibition of NET formation reduces tumor metastasis and IBD occurrence. Hence, as with any immunomodulatory approach, balancing the favorable and unfavorable aspects of NETs formation in each specific situation will be critical, and further exploration and understanding of the regulation and balance of NETs induction, inhibition, and degradation of NETs on pharmacological targets of intestinal disease without compromising the patient's immune defenses is imperative. While multiple methods for detecting NETs are available, there are no uniform criteria to directly define the occurrence of NETs, and in the future, identification of markers and other methods to assess the forming of NETs in vivo as biomarkers and targets for therapeutic interventions in different gut-related diseases is essential. Moreover, more signaling pathways and major regulators of NETs are required to be explored in clinical practice in the future so that we can benefit more from their regulation and thus protect the intestine from damage and carcinogenesis.

## Data Availability

Not applicable.

## Abbreviations

ceDNA: Circulating extracellular DNA
cfDNA: Circulation free DNA
CHD: Congenital heart disease
CLP: Cecum ligation and perforation
cNEC: Cardiac NEC
CRC: Colorectal cancer
CTCs: Circulating tumor cells
DIC: Disseminated intravascular coagulation
DSS: Dextran sulphate sodium
EPEC: Enteropathogenic *Escherichia coli*
FFA2R: Free fatty acid 2 receptor
IBD: Inflammatory bowel disease
iNEC: Inflammatory NEC
IR: Ischemia reperfusion
LPS: Lipopolysaccharide
MRSA: Methicillin-resistant *Staphylococcus aureus*
NE: Neutrophil elastase
NEC: Necrotizing enterocolitis
NETs: Neutrophil extracellular traps
NOD: Non-obese diabetic
NOX-2: NADPH oxidase-2
PAD4: Peptidylarginine deiminase 4
ROS: Reactive oxygen species
SCFAs: Short chain fatty acids
STEC: Shiga-like *Escherichia coli*
TLR: Toll-like receptors
TRIF: TIR-domain-containing adapter inducing interferon-β
